# Clinical Utility of the aMAP Score for Predicting Hepatocellular Carcinoma Development in Patients with Chronic Hepatitis B

**DOI:** 10.3390/diagnostics14131325

**Published:** 2024-06-22

**Authors:** Supakorn Chaiwiriyawong, Suraphon Assawasuwannakit, Poorikorn Feuangwattana, Pimsiri Sripongpun, Naichaya Chamroonkul, Teerha Piratvisuth, Apichat Kaewdech

**Affiliations:** 1 Division of Internal Medicine, Faculty of Medicine, Prince of Songkla University, Songkhla 90110, Thailand; schaiwiriyawong@gmail.com; 2 Department of Medicine, Panyananthaphikkhu Chonprathan Medical Center, Srinakharinwirot University, Nonthaburi 11120, Thailand; suraphon@g.swu.ac.th; 3 Gastroenterology and Hepatology Unit, Division of Internal Medicine, Faculty of Medicine, Prince of Songkla University, Songkhla 90110, Thailand; poorikorn.f@gmail.com (P.F.); spimsiri@medicine.psu.ac.th (P.S.); naichaya@gmail.com (N.C.); 4 NKC Institute of Gastroenterology and Hepatology, Songklanagarind Hospital, Prince of Songkla University, Songkhla 90110, Thailand; teerha.p@psu.ac.th

**Keywords:** HCC, aMAP score, chronic hepatitis B, surveillance, Thailand

## Abstract

This study aimed to evaluate the efficacy of the aMAP score and compare it with other risk scores for predicting hepatocellular carcinoma (HCC) development in Thai patients with chronic hepatitis B (CHB). We retrospectively analyzed patients with CHB between 1 January 2008 and 31 December 2019. Data on demographics, clinical parameters, cirrhosis status, HCC imaging, and alpha fetoprotein surveillance were collected to calculate the aMAP score (0–100) based on age, sex, albumin–bilirubin level, and platelet count. Of the 1060 patients analyzed, 789 were eligible, of whom 51 developed HCC. The cumulative HCC incidences in the low-, moderate-, and high-risk groups at 3, 5, and 10 years were significantly different (log-rank, *p* < 0.0001). The area under the receiver operating characteristic curves (AUROCs) of the aMAP scores for predicting HCC at 3, 5, and 10 years were 0.748, 0.777, and 0.784, respectively. Among the risk scores, the CU-HCC score had the highest AUROCs (0.823) for predicting 5-year HCC development. The aMAP score is a valuable tool for predicting HCC risk in Thai patients with CHB and can enhance surveillance strategies. However, its performance is inferior to that of the CU-HCC score, suggesting the need for new predictive tools for HCC surveillance.

## 1. Introduction

Liver cancer is a malignancy which is spreading worldwide, including Thailand [1]. Hepatocellular carcinoma (HCC) accounts for 85% of all liver cancers. In 2020, HCC ranked seventh in terms of new cases and was the third leading cause of death among all cancers [2]. The global age-standardized rates (ASRs) were 9.5 and 8.7 per 100,000 person-year for HCC incidence and death, respectively [3]. Male sex showed a 2–3 times higher risk of death and new cases annually [2]. The ASRs of incidence were 14.1 and 5.2 per 100,000 person-year in males and females, respectively. In addition, the ASRs of death were 12.9 and 4.8 per 100,000 person-year in males and females, respectively. Data from the Thai Ministry of Public Health also showed an enormous burden of liver cancer, as it ranked as the most common and the fifth most common cancer in Thai males and females, respectively [4]. The ASRs were 22.6 and 21.9 per 100,000 person-year for the incidence and death, respectively, and have increased by 4.7–7.6% since 2018 [5]. The main causes of HCC in Western countries are hepatitis C virus (HCV) infection and metabolic dysfunction-associated steatotic liver disease (MASLD). In contrast, the main causes of HCC in Eastern countries, including Thailand, are hepatitis B virus (HBV) infection and alcohol consumption [6,7,8].

Over the past few decades, several HCC risk scores have been developed and validated to stratify the risk of HCC development [9,10,11,12]. Nevertheless, the majority of these scoring systems heavily emphasize viral factors and predominantly yield satisfactory results within specific patient groups, especially those with HBV infections, and within certain ethnic demographics, such as Asian and Caucasian. Consequently, the applicability of these risk scores is somewhat restricted in modern medical practice, which is characterized by the widespread achievement of viral suppression or elimination through antiviral therapies.

In 2020, an international, multi-etiological, multi-ethnic, prospective chronic hepatitis cohort study developed and validated the aMAP score for predicting HCC development [13]. The score, which is on a scale from 0 to 100, is calculated using only four parameters: age, male gender, the albumin–bilirubin (ALBI) grade, and platelet count. This model accurately determined HCC risk among patients with diverse hepatitis etiologies and across multiple ethnic groups. The aMAP score showed excellent discrimination and calibration in assessing the 5-year HCC risk among all cohorts.

Currently, there are no studies on the external validation of the aMAP score in patients with chronic hepatitis (CHB) in Thailand. The aMAP score should be ascertained by external validation to identify patients with different HCC risks and individualize HCC surveillance in Thailand. Therefore, this study aimed to evaluate the efficacy of the aMAP score and to compare it with other risk scores for predicting HCC development in Thai patients with CHB.

## 2. Methods

### 2.1. Ethics Statements

This study was conducted in accordance with the ethical guidelines of the 1975 Declaration of Helsinki and approved by the Human Research Ethics Unit (HREU) of the Faculty of Medicine, Prince of Songkla University (REC number: 64-380-14-1). As this was a retrospective analysis of de-identified medical records, the need for informed consent was waived by the HREU.

### 2.2. Study Design

Patients diagnosed with CHB at Songklanagarind Hospital from 1 January 2008 to 31 December 2019 were consecutively screened for eligibility. The inclusion criteria were as follows: (i) hepatitis B surface antigen (HBsAg) positivity for at least 6 months and (ii) age ≥ 18 years at diagnosis. The exclusion criteria were as follows: (i) patients diagnosed with HCC within 6 months of their first visit; (ii) patients whose imaging or blood sampling was performed outside Songklanagarind Hospital; and (iii) patients co-infected with HCV or human immunodeficiency virus.

### 2.3. Data Collection and Follow-Up

All data (demographic, biochemical, virological, histological, and radiological features) were retrospectively extracted from the hospital’s electronic patient database. Baseline data were defined as those at or closest to HBsAg positivity within 6 months. Baseline clinical and laboratory parameters, including age, sex, aspartate aminotransferase (AST), alanine aminotransferase (ALT), albumin, total bilirubin, direct bilirubin, hepatitis B e antigen (HBeAg), HBV DNA, and platelet counts, were collected. The primary outcome was the development of HCC. The follow-up endpoint was the date of HCC diagnosis or last outpatient clinic visit in the absence of HCC development. Patients who were lost to follow-up were censored at the last documented visit.

### 2.4. Diagnoses and Clinical Evaluations

Cirrhosis was defined as any one of the following: (i) liver biopsy showing cirrhosis or (ii) abdominal imaging showing modalities with coarse liver echotexture or nodular, parenchymal, or morphological abnormalities and signs of gastroesophageal varices. HCC was diagnosed on the basis of histological evidence or typical radiological features, such as dynamic computed tomography and/or magnetic resonance imaging findings (nodules > 1 cm with arterial hypervascularity and portal/delayed-phase washout) [6,7,14].

### 2.5. HCC Risk Scores and Cutoff Points for Risk Stratification

Six published HCC risk scores (REACH-B [12], CU-HCC [15], PAGE-B [9], mPAGE-B [10], ALBI [16], and aMAP [13]) based on clinical characteristics and laboratory parameters were used. Patients were then categorized into low-, intermediate-, and high-risk HCC stratification groups according to the cutoff points, as previously described (Table S1).

### 2.6. Statistical Analysis

Continuous variables including age, HBV DNA level, liver function test results, complete blood counts, alpha fetoprotein (AFP) levels, ALBI score, HCC risk scores, and follow-up time are presented as mean ± standard deviation or median (interquartile range) and were compared using the *t*-test or rank-sum test. Frequency variables, including sex, HBeAg, AFP group, and cirrhosis, are expressed as numbers and percentages and were compared using the chi-square test or Fisher’s exact test as appropriate. Univariate and multivariable Cox proportional hazards regression models were used to estimate the effects of various variables on the risk of HCC occurrence. Hazard ratios (HRs) and their 95% confidence intervals (CIs), along with the corresponding *p*-values, are presented. The cumulative probabilities of HCC occurrence at different time points (3, 5, and 10 years) were estimated using the Kaplan–Meier method and compared using the log-rank test. The predictive performance of the aMAP and other scores was assessed using the area under the receiver operating characteristic curve (AUROC). Statistical differences in the AUROC between aMAP and the other scores were evaluated using the Delong test. The Harrell concordance index (C-index) and Akaike Information Criterion (AIC) were used to assess the score discrimination ability. To assess the diagnostic accuracy of the suggested cutoff points of the aMAP score, sensitivity, specificity, and positive (PPV) and negative (NPV) predictive values were calculated at the given cutoff points. Statistical analyses were performed using R software (version 4.2.3; R Core Team, Vienna, Austria). Two-sided *p*-values < 0.05 were considered to be statistically significant.

## 3. Results

### 3.1. Baseline Clinical Characteristics

**Figure 1:**
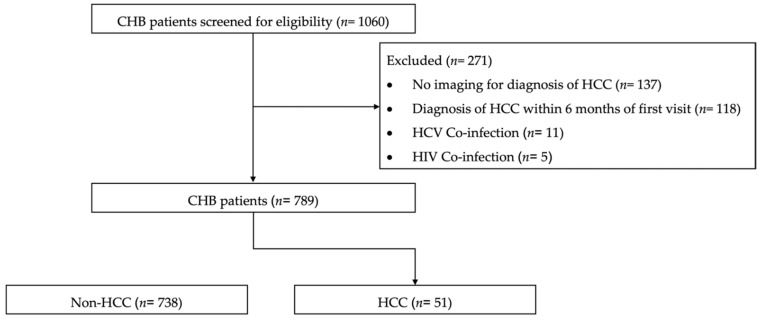
Flowchart of our cohort. CHB, chronic hepatitis B; HCC, hepatocellular carcinoma; HCV, hepatitis C virus; HIV, human immunodeficiency virus.

We identified 1060 patients with CHB; 271 patients were excluded according to the exclusion criteria, the majority due to the absence of hepatic imaging or pathology indicating HCC development and HCC diagnosis within 6 months of the baseline visit. Finally, 789 patients with CHB were analyzed (Figure 1).

Patients’ baseline characteristics are presented in Table 1. Patients with HCC were older than those without HCC (median age: 56.1 years versus [vs.] 54.2 years, *p* = 0.02), and there was a higher proportion of male patients with HCC than male patients without HCC (68.6% vs. 50.3%, *p* = 0.02). Liver biochemistry tests, including AST, ALT, total bilirubin, and direct bilirubin levels, were significantly higher in patients with HCC than in those without. However, serum albumin levels and platelet counts were lower in patients with HCC than in those without. Similarly, serum AFP levels were significantly higher in patients with HCC than in those without. A very high proportion of patients with HCC had cirrhosis (94.1%) compared to those without (28.2%). During the follow-up period, a significantly higher proportion of patients with HCC used nucleotide or nucleoside analogs than those without (100% vs. 49.5%; *p* < 0.001).

Among the risk scores (ALBI, aMAP, REACH-B, CU-HCC, PAGE-B, and mPAGE-B), all of these risk scores were significantly different between the two groups, with patients with HCC generally having higher or more scores than those without.

### 3.2. Cumulative Incidence of HCC Development in the Entire Cohort

**Figure 2:**
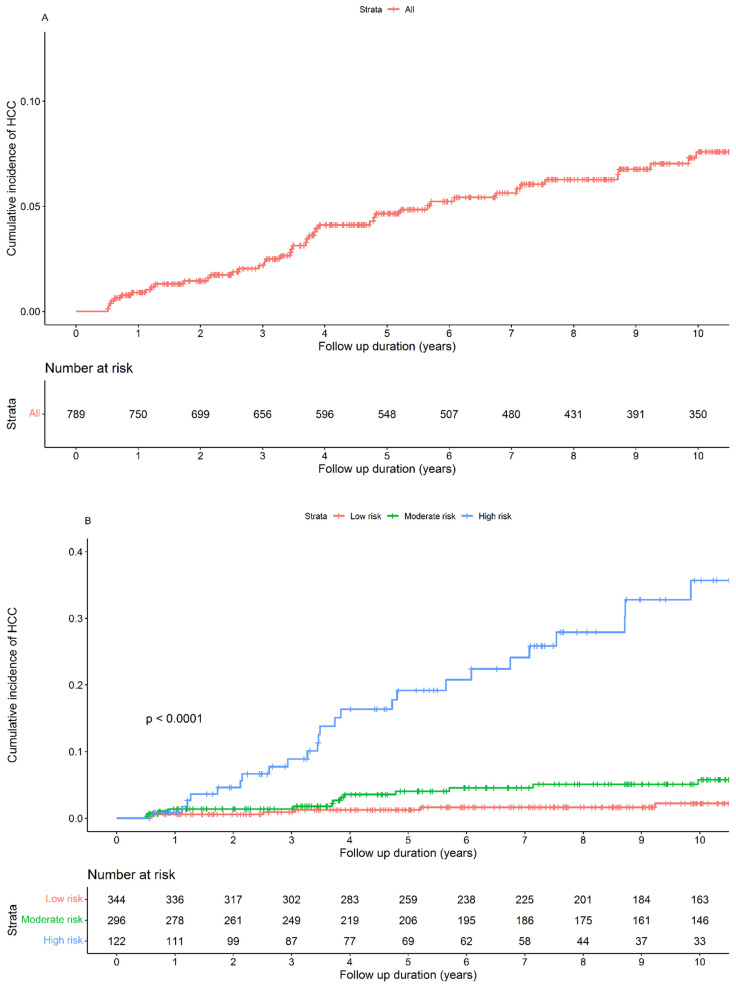
Cumulative probability of HCC development. (**A**) Complete cohort. (**B**) Risk stratification by the aMAP score. HCC, hepatocellular carcinoma.

The cumulative incidences of HCC in the cohort at 1, 3, 5, and 10 years were 0.89%, 2.17%, 4.55%, and 7.32%, respectively (Figure 2A). When stratified by the diagnosis of CHB, we found that the cumulative incidences of HCC at 1, 3, 5, and 10 years for CHB diagnoses before 2014 were 0.60%, 2.04%, 4.41%, and 7.32%, respectively, and for diagnoses after 2014, they were 1.43%, 2.28%, 4.72%, and 5.61%, respectively.

### 3.3. Cumulative Incidence of HCC Development According to the aMAP Score

When we applied the aMAP score to our cohort, 45.1% were categorized as low-risk, followed by 38.8% and 16.1% in the moderate- and high-risk groups, respectively. By stratifying the aMAP score (Figure 2B), the cumulative incidences of HCC in the low-risk group at 1, 3, 5, and 10 years were 0.58%, 0.91%, 1.24%, and 2.2%, respectively. In the moderate-risk group, the cumulative incidence rates were 1.38%, 1.38%, 3.96%, and 5.61%, respectively. In the high-risk group, the cumulative incidences were 0.83%, 8.51%, 17.44%, and 30.01%, respectively. The risk between the three groups was significantly different (*p* < 0.0001). We also analyzed the patients diagnosed with CHB before and after 2014 and found that the aMAP score can discriminate between the different HCC risk groups (Figure S1A,B). Before 2014, the cumulative incidences of HCC according to the aMAP score were as follows: in the low-risk group, 0%, 0.47%, 0.94%, and 1.53%; in the moderate-risk group, 1.00%, 1.00%, 3.17%, and 4.99%; and in the high-risk group, 1.41%, 10.38%, 20.11%, and 35.25%. After 2014, the cumulative incidences of HCC according to the aMAP score were as follows: in the low-risk group, 1.58%, 1.58%, 1.58%, and 3.44%; in the moderate-risk group, 2.20%, 2.20%, 6.51%, and 6.51%; and in the high-risk group, 0%, 5.14%, 12.16%, and 12.16%.

### 3.4. Comparison of the Predictive Performance of the aMAP Score and Other Existing HBV-Related HCC Risk Scores

The performance and discrimination of the aMAP score and other scores (ALBI, REACH-B, CU-HCC, PAGE-B, and mPAGE-B) were compared (Table 2). The AUROCs predicting HCC at 3, 5, and 10 years for the aMAP score were 0.748, 0.777, and 0.784, respectively. The 5- and 10-year AUROC values for the CU-HCC score were slightly higher than those of the aMAP score (0.823 [*p* = 0.250] and 0.830 [*p* = 0.216], respectively). Additionally, the discriminatory ability of the CU-HCC score calculated using the C-index demonstrated the highest value of 0.826. The C-indexes of the aMAP score, REACH-B, PAGE-B, and mPAGE-B were 0.766, 0.696, 0.737, and 0.732, respectively.

The sensitivity, specificity, PPV, and NPV of the aMAP score for predicting HCC were evaluated further (Table 3). The aMAP model cutoff points of 50 and 60 had NPVs of 98% and 96% in predicting HCC, respectively.

### 3.5. Predictors of HCC Development

**Figure 3:**
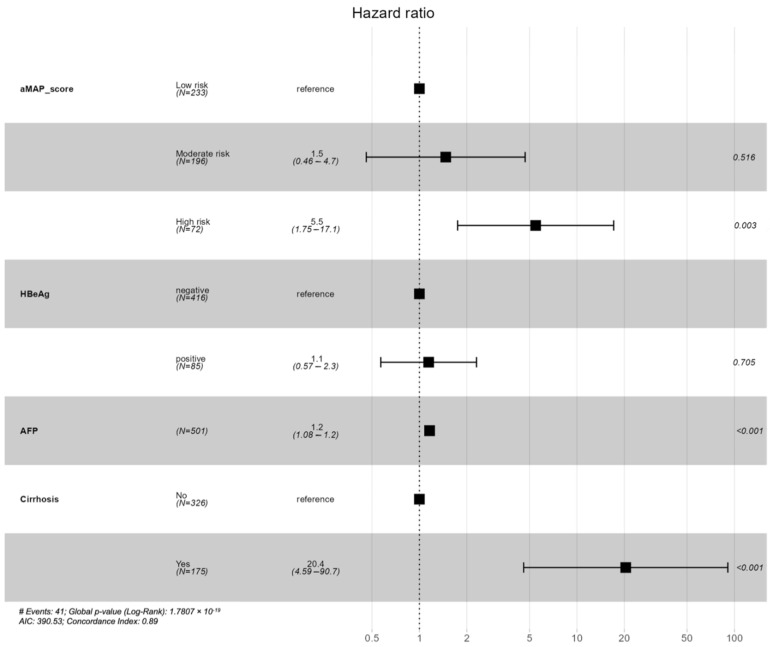
Forest plot of factors associated with HCC development. HCC, hepatocellular carcinoma; HBeAg, hepatitis B e antigen; AFP, alpha fetoprotein; AIC, Akaike information criterion.

We further evaluated the predictors of HCC development and confirmed the utility of the aMAP score in predicting HCC. Multivariable analysis confirmed that the aMAP score, particularly in the high-risk group, was an independent predictor of HCC development after adjusting for AFP level, presence of cirrhosis, and HBeAg status (Figure 3). The HRs for moderate risk and high risk were 1.5 (95% CI: 0.46–4.7, *p* = 0.516) and 5.5 (95% CI: 1.75–17.1, *p* = 0.003), respectively. We also explored other predictors, such as AFP level, cirrhosis status, and treatment received, alongside the aMAP score. We found that a high-risk aMAP score remained an independent risk factor for HCC development (adjusted HR 4.78 [95% CI: 1.7–13.41], *p* = 0.003).

## 4. Discussion

Our study confirmed the clinical utility of the aMAP score to predict HCC development among patients with CHB. However, the HCC prediction performance was not significantly different from that of the other predictive scores. The aMAP score tended to predict the 5- and 10-year risk of HCC better than the other scores, except for the CU-HCC score.

Our cohort demonstrated a cumulative HCC incidence of 6.46%, with a median follow-up time of >8.9 years. Our HCC incidence was similar to those of other Asian studies, which ranged from 3.7% to 10.4% [12,15,17,18]. The HBV genotype was similar to genotypes B and C [19]. In contrast to Caucasian studies [9], which reported the incidence of HCC to be 3.5%, the incidence in our cohort was higher because of the different HBV genotypes, with more prevalent genotypes A and D [20]. HBV genotypes play a role in the aggressiveness of HCC development, with genotype C contributing to HCC in >25% of patients [21].

We then explored the HCC risk stratification by the aMAP score. The aMAP score can discriminate the patients into three risk categories with a significant difference among them. Most patients were categorized as low-risk (45.1%), with a 10-year cumulative HCC incidence of approximately 1.74%. On the contrary, the high-risk aMAP score showed a cumulative incidence of HCC up to 20% at 10 years. Our results confirmed the findings of the original cohort.

The performance of the aMAP score for 5-year HCC risk prediction was numerically inferior to that of the CU-HCC score. This finding was different from the original study, with the AUROC of the aMAP score being higher than that of the CU-HCC score (AUROC, 0.82–0.87 vs. 0.68–0.85) [13]. We further analyzed the 10-year HCC risk by extending it to the original value. The aMAP score showed a moderate performance, but this was not statistically different from the performance of other scores. These findings can be used to predict the long-term risk of HCC in patients with CHB. Furthermore, the CU-HCC score showed the best performance across all time points. The formula for the CU-HCC score was designed exclusively for CHB with additional factors, including HBV DNA levels and the presence of cirrhosis, for risk calculation [15]. The aMAP score includes age, male gender, the ALBI grade, and platelet count and does not include cirrhosis and HBV viral load, which are incorporated in the CU-HCC score. Cirrhosis is strongly associated with the risk of developing HCC. More than 80% of HCC cases occurred in patients with cirrhosis [22]. Similarly, an increase in HBV DNA levels is linked to HCC risk [23].

The aMAP score had a high NPV value of 96–98%, as in the original study [13]. The aMAP score could be beneficial in at-risk patients and for categorizing the low-risk group with a score <50 or 60 from the moderate- or high-risk group. Patients with CHB with indications for HCC surveillance can undergo HCC surveillance with liver ultrasonography, with or without AFP surveillance, every 6 months. However, the moderate- or high-risk aMAP score might require other surveillance tools to increase the rate of early HCC detection. Liver ultrasonography may be of inadequate quality in one-fifth of patients, particularly in those with obesity [24].

Finally, we performed multivariable Cox regression analyses to determine the factors associated with HCC prediction. We found that the aMAP score was an independent risk factor for HCC development after adjusting for HBeAg status, AFP level, and the presence of cirrhosis. The finding supported the utility of the aMAP score. Additionally, an increase in AFP levels and the presence of cirrhosis at baseline had an impact on HCC prediction, with the strongest risk factor being cirrhosis, with an HR of 20. These two factors can be explored and incorporated to develop an updated version of the aMAP score or a new HCC prediction model.

Our study has many strengths. First, to our best knowledge, this is the first study that externally validated the usefulness of the aMAP score in Thai patients with CHB. Second, we validated the score in real-world patients with CHB and not in a clinical trial, like that conducted in the originally developed cohort, both with and without antiviral treatment. In this trial, all patients received antiviral agents during the follow-up period. In contrast, approximately 47% of the patients did not receive an antiviral agent in our study. Treatment could be a factor that modifies the natural history of patients at the beginning when applying the risk score [25].

We also acknowledge the limitations of this single-center study and that it was conducted only in Asian countries. Therefore, our findings may not apply to other populations. Additionally, our center is a referral center, and patient characteristics may differ from those of general patients with CHB. Further studies will be conducted to update the aMAP score by incorporating other significant factors, including AFP levels and cirrhosis status, and to validate it externally across multiple centers.

## 5. Conclusions

The aMAP score is a valuable tool for predicting HCC risk in Thai patients with CHB and can enhance surveillance strategies. Nonetheless, its performance is inferior to that of the CU-HCC score, suggesting the need for new predictive tools for HCC surveillance.

## Figures and Tables

**Table 1:** Baseline and on-treatment characteristics of the entire cohort.

Variable	Non-HCC (*n* = 738)	HCC (*n* = 51)	Total (*n* = 789)	*p*-Value
Age, median (IQR), years	54.2 (47.7, 61.7)	56.1 (53.1, 65.5)	54.6 (47.8, 61.9)	0.02
Male sex, *n* (%)	371 (50.3)	35 (68.6)	406 (51.5)	0.02
Laboratory result				
HBeAg negativity, *n* (%)	486 (83.2)	30 (71.4)	516 (82.4)	0.084
HBV DNA, median (IQR), (IU/mL)	1691.2 (112, 50,008)	2161 (112, 2,120,883)	1691.2 (112, 56,560)	0.216
Total bilirubin, median (IQR), (mg/dL)	0.5 (0.4, 0.8)	0.8 (0.5, 1)	0.6 (0.4, 0.8)	<0.001
Direct bilirubin, median (IQR), (mg/dL)	0.2 (0.1, 0.3)	0.3 (0.2, 0.4)	0.2 (0.1, 0.3)	<0.001
AST, median (IQR), U/L	26 (21, 39)	42.5 (32, 63.8)	27 (21, 42)	<0.001
ALT, median (IQR), U/L	25 (17, 43)	35 (23, 56.5)	25 (17, 43.2)	0.002
Albumin, median (IQR), (g/dL)	4.4 (4.2, 4.6)	4.2 (3.8, 4.5)	4.4 (4.1, 4.6)	<0.001
WBCs, median (IQR), ×10^3^/mm^3^	6.2 (5.1, 7.6)	5.7 (4.9, 6.8)	6.2 (5.1, 7.5)	0.071
Hb, median (IQR), (g/dL)	13.4 (12.3, 14.4)	13.9 (12.3, 14.6)	13.4 (12.3, 14.5)	0.657
Platelet, median (IQR), ×10^3^/mm^3^	216 (177, 260)	147 (101, 198)	213 (171, 258)	< 0.001
AFP, median (IQR), (ng/mL)	2.9 (2, 4.1)	9.2 (3.7, 29.8)	3 (2, 4.5)	<0.001
Cirrhosis, *n* (%)	208 (28.2)	48 (94.1)	256 (32.4)	<0.001
Predictive score				
ALBI, median (IQR)	−3.1 (−3.3, −2.9)	−2.8 (−3.1, −2.4)	−3.1 (−3.3, −2.9)	<0.001
aMAP, mean (SD)	50.7 (8.7)	59 (8.5)	51 (8.9)	<0.001
REACH-B, median (IQR)	8 (6, 10)	10 (8.5, 12)	8 (7, 10)	<0.001
CU-HCC, median (IQR)	4 (3, 18)	18.5 (18, 22)	4 (3, 18)	<0.001
PAGE-B, median (IQR)	7 (3, 11)	13 (8, 17)	7 (3, 11)	<0.001
mPAGE-B, median (IQR)	10 (8, 12)	13 (11, 14)	10 (9, 12)	<0.001
On-treatment				
NUC use, *n* (%)	365 (49.5)	51 (100)	416 (52.7)	<0.001
- Lamivudine, 150 mg	272 (36.9)	35 (68.6)	307 (38.9)	
- Adefovir dipivoxil, 10 mg	5 (0.7)	1 (2)	6 (0.8)	
- Tenofovir disoproxil, 300 mg	7 (0.9)	0 (0)	7 (0.9)	
- Entecavir, 0.5 mg	61 (8.3)	14 (27.5)	75 (9.5)	
- Telbivudine, 600 mg	10 (1.4)	0 (0)	10 (1.3)	
- Lamivudine, 100 mg	3 (0.4)	0 (0)	3 (0.4)	
- Tenofovir alafenamide, 25 mg	7 (0.9)	1 (2)	8 (1)	
No NUC use, *n* (%)	373 (50.5)	0(0)	373 (47.3)	
Follow-up time, median (IQR), years	9.2 (4.5, 12.4)	3.8 (2.3,7.3)	8.9 (4.1, 12.3)	<0.001

ALBI, albumin–bilirubin; AFP, alpha fetoprotein; ALT, alanine aminotransferase; AST, aspartate aminotransferase; Hb, hemoglobin; HBeAg, hepatitis B e antigen; HBV, hepatitis B virus; IQR, interquartile range; NUC, nucleotide or nucleoside analog; WBCs, white blood cells.

**Table 2:** Comparison of the performance between the aMAP score and other predictive scores.

Score	3 y AUROC (95% CI)	*p*-Value *	5 y AUROC (95% CI)	*p*-Value *	10 y AUROC (95% CI)	*p*-Value *	C-Index (95% CI)	AIC
aMAP	0.748 (0.610–0.887)	reference	0.777 (0.686–0.869)	reference	0.784 (0.708–0.860)	reference	0.766 (0.761–0.771)	638.439
ALBI	0.820 (0.736–0.895)	0.342	0.710 (0.611–0.806)	0.203	0.710 (0.622–0.789)	0.059	0.686 (0.680–0.692)	657.163
PAGE-B	0.717 (0.579–0.856)	0.331	0.750 (0.655–0.842)	0.214	0.760 (0.678–0.842)	0.179	0.737 (0.731–0.742)	654.338
mPAGE-B	0.714 (0.585–0.843)	0.193	0.759 (0.669–0.849)	0.293	0.753 (0.679–0.827)	0.039	0.732 (0.727–0.737)	660.076
REACH-B	0.571 (0.441–0.701)	0.023	0.720 (0.633–0.808)	0.105	0.712 (0.629–0.795)	0.027	0.696 (0.689–0.702)	415.170
CU-HCC	0.804 (0.753–0.854)	0.296	0.823 (0.775–0.871)	0.250	0.830 (0.791–0.870)	0.216	0.826 (0.823–0.829)	492.898

AIC, Akaike information criterion; ALBI, albumin–bilirubin; AUROC, area under the receiver operating characteristic curve; C-index, Harrell concordance index. * *p*-value for comparison with the aMAP score.

**Table 3:** Predictive values of the aMAP score for predicting HCC risk at the different cutoff points.

Cutoff Point	Sensitivity, % (95% CI)	Specificity, % (95% CI)	PPV, % (95% CI)	NPV, % (95% CI)
50	84 (71, 93)	48 (44, 52)	10 (8, 14)	98 (96, 99)
60	53 (38, 67)	87 (84, 89)	22 (15, 31)	96 (94, 98)

NPV, negative predictive value; PPV, positive predictive value; CI, confidence interval.

## Data Availability

The data that support the findings of this study are available on request from the corresponding author.

## Supplementary Materials

The following supporting information can be downloaded at: https://www.mdpi.com/article/10.3390/diagnostics14131325/s1, Table S1: Summary of predictive hepatocellular carcinoma scores used in patients with chronic hepatitis B; Figure S1: Cumulative incidence of HCC development according to the aMAP score, stratified by CHB diagnosis before (Figure S1A) and after 2014 (Figure S1B).
