# Supplementary material for: Clinical Utility of the aMAP Score for Predicting Hepatocellular Carcinoma Development in Patients with Chronic Hepatitis B
Source: Diagnostics (Basel). 2024 Jun 22;14(13):1325. doi: 10.3390/diagnostics14131325 (PMC11240693; doi:10.3390/diagnostics14131325)
Supplement: Supplementary file 1 [file diagnostics-14-01325-s001.zip › diagnostics-3057669-supplementary.pdf]

Clinical Utility of the aMAP Score for Predicting Hepatocellular Carcinoma Development in Patients with Chronic Hepatitis B

Supakorn Chaiwiriyawong [1], Suraphon Assawasuwannakit [2], Poorikorn Feuangwattana [3], Pimsiri Sripongpun [3], Naichaya Chamroonkul [3], Teerha Piratvisuth [4] and Apichat Kaewdech [1,*]

[1] Division of Internal Medicine, Faculty of Medicine, Prince of Songkla University, Songkhla, Thailand
[2] Department of Medicine, Panyananthaphikkhu Chonprathan Medical Center, Srinakharinwirot University, Nonthaburi, Thailand
[3] Gastroenterology and Hepatology Unit, Division of Internal Medicine, Faculty of Medicine, Prince of Songkla University, Songkhla, Thailand
[4] NKC Institute of Gastroenterology and Hepatology, Songklanagarind Hospital, Prince of Songkla University, Songkhla, Thailand
* Correspondence: Apichat Kaewdech, Gastroenterology and Hepatology Unit, Division of Internal Medicine, Faculty of Medicine, Prince of Songkla University, Hat Yai 90110, Thailand; Tel: +6674-451479

Risk score	Calculation	Cut-off point
**aMAP**	((0.06×age+0.89×sex (Male: 1, Female: 0)+0.48×((log10bilirubin×0.66)+(albumin×-0.085))–0.01×platelets)+7.4)/14.77×100, age is in years, bilirubin in μmol/L, albumin in g/L and platelets in 103/mm^3	Low-risk: <50<br>Medium-risk: 50–60<br>High-risk: >60
**REACH-B**	Male sex: 2 points+Age: 30–34 (0 points), 35–39 (1 point), 40–44 (2 points), 45–49 (3 points), 50–54 (4 points), 55–59 (5 points), 60–65 (6 points)+ALT (IU/L): 15–<45 (1 point), ≥45 (2 points)+ HBeAg positivity: 2 points+HBV DNA copies/mL <9999 (0 points),10,000–99,999 (3 points), 100,000–999,999 (5 points), >1,000,000 (4 points)	Low-risk: 0–8<br>Medium-risk: 9–11<br>High-risk: 12–17
**CU-HCC**	Age (>50 y=3; ≤50=0)+albumin (≤3.5=20; >3.5=0)+bilirubin (>1=1.5; ≤1=0)+HBV DNA (<4 log copies/mL=0; 4–6=1; >6=4)+cirrhosis (yes=15; no=0)	Low-risk: <5<br>Medium-risk: 5–19<br>High-risk: ≥20
**PAGE-B**	Sex (male=6; female=0)+age (16–29 years=0; 30–39 years=2; 40–49 years=4; 50–59 years=6; 60–69 years=8; ≥70 years=10+platelet (≥200,000/mm^3=0; 100,000–199,999/mm^3=6; ≤100,000/mm^3=9)	Low-risk: <10<br>Medium-risk: 10–17<br>High-risk: >17

**mPAGE-B**	Sex (male=2; female=0)+age (<30 years=0; 30–39 years=3; 40–49 years=5; 50–59 years=7; 60–69 years=9; ≥70 years=11)+platelet (≥250 =0; 200–250=2; 150–200=3; 100–150=4; <100=5)+albumin (≥4.0=0; 3.5–4.0=1; 3.0–3.5 =2; <3=3), albumin is in g/dL and platelets in 103/mm^3	Low-risk: <9<br>Medium-risk: 9–12<br>High-risk: >12
**Albumin-Bilirubin (ALBI) score**	($\log_{10}$ bilirubin [μmol/L] × 0.66) + (albumin [g/L] × −0.0852)	ALBI grade 1: score ≤ −2.60<br>ALBI grade 2: > −2.60 to ≤ −1.39<br>ALBI grade 3: > −1.39

Table S1. Summary of predictive hepatocellular carcinoma scores used in patients with chronic hepatitis B

ALT, alanine aminotransferase; HBeAg, hepatitis B e antigen; HBV, hepatitis B virus

A

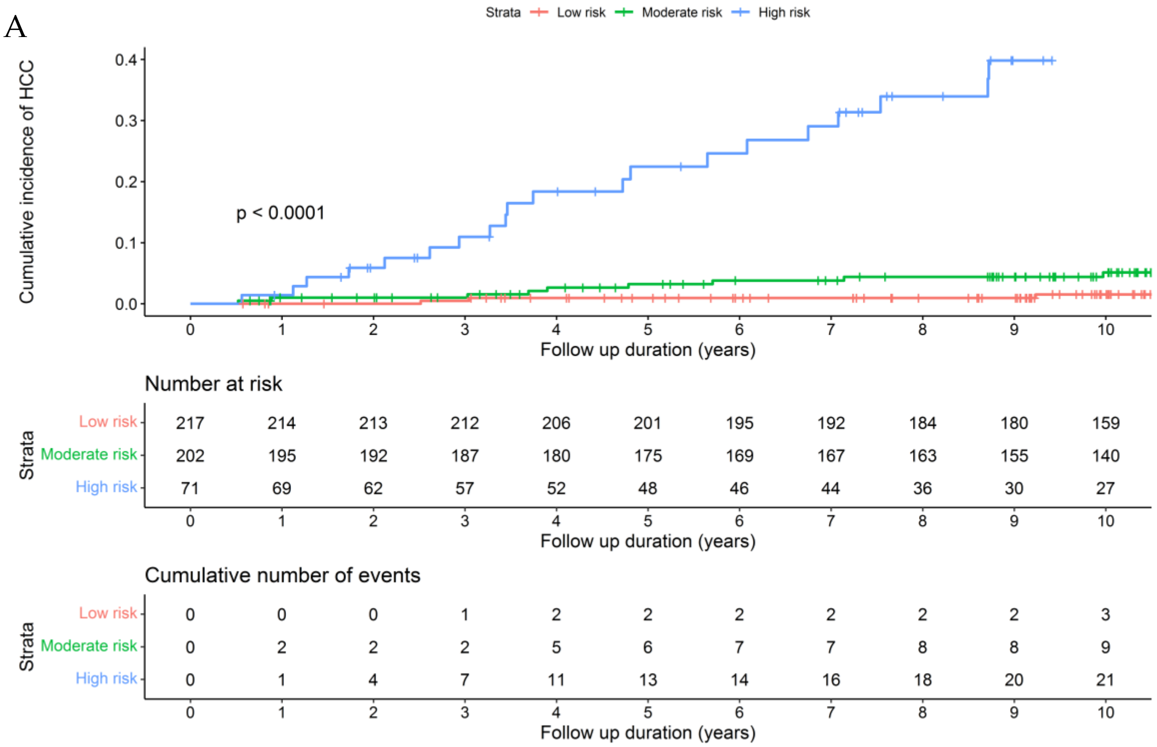

B

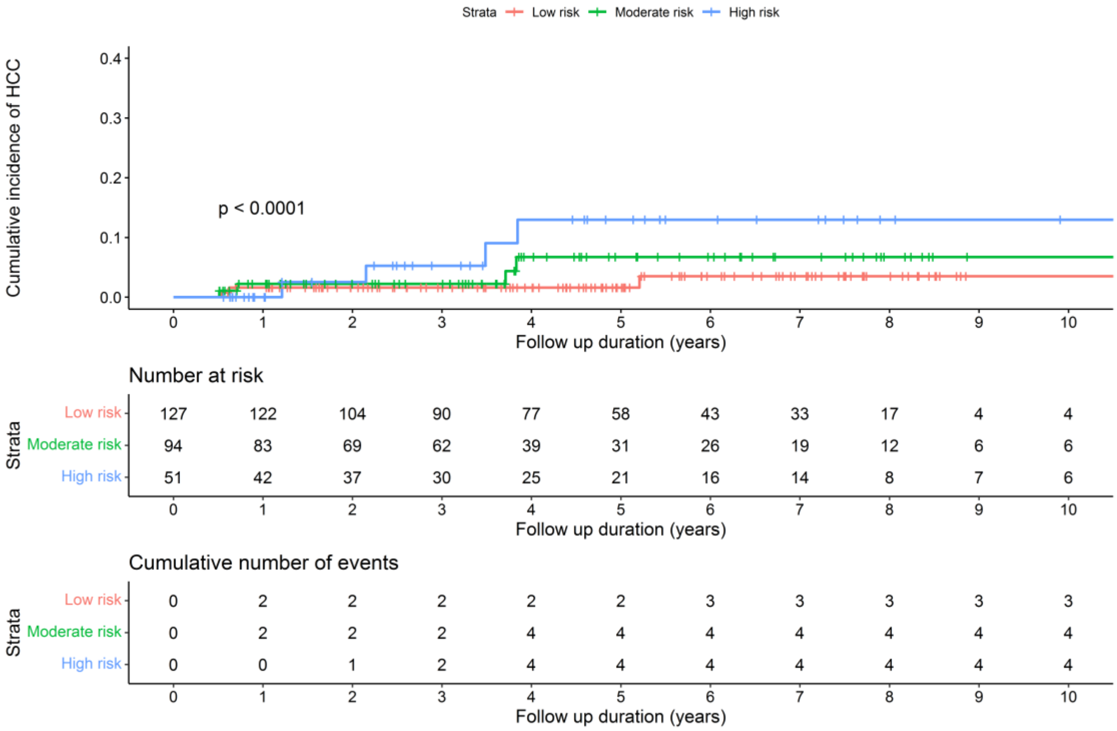

Figure S1: Cumulative incidence of HCC development according to the aMAP score, stratified by CHB diagnosis before (Figure S1A) and after 2014 (Figure S1B).
